# Impact of home-visit counselling on maternal and child health and nutrition by Barangay health workers: a quasi-experimental study from Eastern Visayas, Philippines

**DOI:** 10.7189/jogh.15.04276

**Published:** 2025-09-26

**Authors:** Yunhee Kang, Ahreum Choi, Heunghee Kim, Anbrasi Edward, Heyeon Ji, Jihwan Jeon

**Affiliations:** 1 Johns Hopkins School of Public Health, Department of International Health, Maryland, USA; 2 Seoul National University, Department of Food and Nutrition, Seoul, South Korea; 3 Seoul National University, Graduate School of Public Health, Seoul, Republic of Korea; 4 Seoul National University, Research Institute of Human Ecology, Seoul, Republic of Korea; 5 World Vision Korea, Seoul, Republic of Korea

## Abstract

**Background:**

Maternal and child undernutrition and poor mental health remain public health concerns in the Philippines. We evaluated the impact of home visits with timed and targeted care for family (ttCF) strategies for maternal and child nutrition and mental health during the first 1000 days of life.

**Methods:**

We designed a quasi-experimental study encompassing 12 municipalities (six intervention and six comparison) and enrolling 1518 pregnant women or mothers with children <12 months. Trained Barangay health workers (BHWs) conducted 12 scheduled home visits from pregnancy through child’s second year in the intervention areas, while comparison areas received routine health services. We estimated the intervention’s impact using multilevel mixed-effect models and generalised linear models, adjusting for socioeconomic covariates. The outcomes of interest included frequency of BHW’s home visits, maternal message recall scores, possible depressive symptoms (Edinburgh Postnatal Depression Scale (EPDS) score >9), maternal and child diet, and child nutritional status.

**Results:**

We assessed 1313 women and children (655 in comparison; 658 in intervention) at 12-month follow-up. Women in the intervention areas received higher home visits during pregnancy (mean (x̄) = 3.2, standard deviation (SD) = 2.7 *vs*. x̄ = 1.7, SD = 2.0; *P* < 0.001) and postpartum (x̄ = 2.6, SD = 2.1 *vs*. x̄ = 1.3, SD = 1.4; *P* < 0.001), and they had higher recall of health and nutrition messages across the intervention period: prenatal care (adjusted mean difference (AMD) = 2.25; 95% CI = 1.57, 2.94), birth (AMD = 1.83; 95% CI = 1.24, 2.41), and postpartum care (AMD = 2.20; 95% CI = 1.43, 2.96). The intervention reduced maternal potential depressive symptoms by 9.16 percentage points (95% CI = −15.7, −2.62) and a reduction of 1.20 points in EPDS score (95% CI = −1.88, −0.52). There were no significant differences between intervention and comparison groups for maternal dietary diversity or child diet and nutrition indicators.

**Conclusions:**

The ttCF strategy improved the frequency and quality of BHW home visits and demonstrated promising impacts on maternal mental health. However, additional enabling interventions are needed to improve maternal and child diet and nutrition outcomes.

Despite ongoing national efforts, maternal and child undernutrition remains an unaddressed public health challenge in the Philippines. Nearly one out of three children under five years of age is stunted (29%), exceeding the Asia region average of 21.8% [1], while one in four pregnant women (26.6%) is anaemic [2]. The diet quality of young children is poor; only 37.3% meet the minimum standard of dietary diversity and feeding frequency [1].

Maternal mental health during pregnancy and postpartum plays a crucial role in shaping both maternal and child health outcomes. Depression in these periods leads to poor obstetric outcomes, low birth weight, preterm delivery, and impaired physical health and cognitive development in infants [3–5]. Depressed mothers may impair their caregiving capacity, leading to sub-optimal breastfeeding and complementary feeding practices that further exacerbate the risk of undernutrition [6].

Evidence has shown that community-based interventions, including home-based counselling by community health workers (CHWs), can enhance maternal knowledge and behaviours around health and nutrition and improve health outcomes when paired with clinical services [7,8]. In the Philippines, Barangay health workers (BHWs), officially recognised under Republic Act No. 7883, provide primary health services in rural areas [9,10]. Despite their vital contributions to the health system, BHWs face resource limitations, while decentralisation has led to regional disparities in training, compensation, and supervision, undermining the effectiveness of their activities in addressing maternal and child health challenges [11].

A maternal, newborn, and child health (MNCH) project was implemented in the Eastern Visayas, Philippines, in 2021 and is ongoing [12]. Its goal is to enhance the utilisation of prenatal and postpartum services and to improve the health and nutrition outcomes for pregnant and lactating women and young children. The MNCH project also supports birthing facility licenses, enhanced the capacity of health care workers, and raised community awareness of health. The Timed and Targeted Care for Family (ttCF) programme [13], as a key MNCH component, is localised to the context, emphasising family participation and gender equality to improve maternal and child health during the first 1000 days.

The objective of this study was to evaluate the impact of the timed and targeted counselling BHW home visits in the ttCF programme in Eastern Visayas. Outcome measures of interest were frequency and content of home visits, maternal mental health, maternal and child dietary diversity, and nutritional status.

## METHODS

### Study setting

The MNCH project was conducted in 16 municipalities across four provinces-Northern Samar, Western Samar, Eastern Samar, and Leyte-in the Eastern Visayas. Approximately 90% of the population is Roman Catholic, but traditional Filipino beliefs coexist [14]. The incidence of poverty among families in Eastern Samar was estimated at 24.9% in 2023 [15]. The maternal mortality ratio in Eastern Visayas (139 deaths per 100 000 live births) is above the national average (54 deaths per 100 000 live births) [16]. The region has an inadequate health care infrastructure, which contributes to a high rate of home births without skilled attendance, while limited access to postpartum care means many mothers lack appropriate medical support after childbirth [1].

### Timed and targeted care for family

Timed and Targeted Counselling (ttC) is a community health programme for mothers and children based on a family-centred behaviour change communication strategy [17]. It includes various topics related to pregnancy, delivery, postnatal care, and early childhood childcare, inclusive of breastfeeding and complementary feeding across the first 1000 days of life and communication skills.

The ttC approach was adapted into the ttCF through a partnership between the MNCH team and the Department of Health – Philippines. The ttCF introduced several key modifications: Barangay nutrition scholars were involved alongside BHWs in conducting home visits; the ttCF messaging incorporated the eight or more antenatal care (ANC) visits recommended by the World Health Organization (WHO); the postnatal care period was extended to 45 days after delivery, compared to the previous standard of 42 days. A ttCF monitoring tool that includes household registration and database construction was developed through a collaborative effort by experts from different sectors. The process of developing the ttCF and details on field supervision and monitoring are described elsewhere [12].

In total, 1865 BHWs received competency training on timely messages and counselling skills in their respective areas in June 2023, including 502 BHWs in Eastern Samar and 496 BHWs in West Samar. In December 2024, a total of 6280 households were registered for ttCF visits. The BHWs filled out ttCF forms during home visits and later submitted it to nurses at rural health units (RHUs), who then delivered them to an MNCH field facilitator for data checks and entry into an Excel-based programme. Some RHUs actively provided oversight for the BHW home visit and validated programme operations at the local level. The RHU staff collaborated with the MNCH project team and municipal health offices to review the quality and timeliness of BHW visits, participation of household members, content of discussions, rapport building, use of ttCF tools, and client satisfaction.

### Study design and intervention allocation

We designed the evaluation research as a quasi-experimental study with 12 municipalities, *i.e.* six comparison municipalities (Giporlos, Oras, and San Julian from Eastern Samar and Pinabacdao, Hinabangan, and San Sebastian from Western Samar) and six intervention municipalities (Taft, General MacArthur, Quinapondan from Eastern Samar and Marabut, Basey, and San Jorge from Western Samar).

Study areas were selected through iterative discussions among the research team, the MNCH project team, and local government representatives. Out of four MNCH project provinces, Northern Samar was excluded due to a far distance from the project office in Tacloban City, Leyte (approximately 291km), while Leyte was not prioritised due to the high accessibility to RHUs and a relatively small number of pregnant women (n = 615). In contrast, Western and Eastern Samar had larger populations of pregnant women with livebirths, 1011 and 1268, respectively (internal records), and only low to medium RHU accessibility.

Intervention municipalities within these provinces were chosen as project priorities, as they had a relatively high number of pregnant women. Municipalities with many geographically isolated and disadvantaged areas or ongoing security concerns were excluded. Preference was given to municipalities where local governments and health officials were reportedly more cooperative, making them more suitable for study.

In the intervention areas, BHWs who were trained by ttCF conducted scheduled home visits from July 2023 onwards for pregnant women and mothers with children aged 0–23 months: during pregnancy (four visits), from birth to six months of age (four visits), and thereafter to 24 months after birth (four visits). The BHWs conducted the following activities: defining and mapping the catchment area; conducting community sensitisation with key stakeholders; registering all families within the designated zone; identifying pregnancies and children under two; conducting scheduled and timed home visits; engaging families in dialogue using storybooks; monitoring and providing follow-up support using the Household Handbook [18]; referring emergency cases to health facilities; reporting to supervisor every 1–3 months; The counselling messages included timely health and nutrition topics related to pregnancy, childbirth, postpartum care, and infant and child health and nutrition by the timing of visit (Table S1 in the **Online Supplementary Document**). The BHWs marked which messages were delivered to the registered participants using the checklists, as well as documented the health practices adhered to by each household. For health practices not done by the household, negotiated agreements were established and recorded. The BHWs were provided with non-monetary compensation for household visits.

The BHWs in comparison areas provided routine services as mandated by the government, such as antenatal, delivery, and post-natal health services.

### Study participants, recruitment, and sample size

We included women aged 15–49 years, either pregnant or having one or more children aged 0–11.9 months at the time of enrolment, and followed them up after a year.

To test an 8% difference in minimum dietary diversity among children aged 6–23 months after one year of follow-up, we estimated we would require a sample size of 551 per group. Factoring in a 15% loss to follow-up and 1.2 design effect, the required sample size increased to 760 women per group, *i.e.* a total of 1520 participants. To obtain this sample, we aimed to recruit an average of 127 participants from each 12 municipalities according to the probability proportional to size approach [19].

### Outcomes of interest

**Figure 1:**
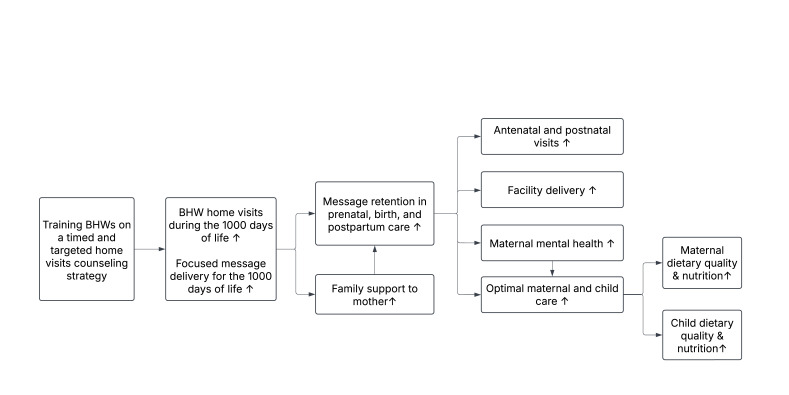
Conceptual framework.

Outcomes of interest were determined along the potential programme impact pathway (**Figure 1**). Study women were asked about the frequency of home visits by BHWs during pregnancy, since their delivery, and until the time of the survey visit, as well as about any attendance with family members during BHW’s visits.

We defined the percentage of adherence of BHW home visits as the number of BHW home visits received over the past year divided by the recommended number of visits based on the child’s current age, including pregnancy at the time of enrolment. The expected number of visits in the past year was as follows: three visits for children aged ≥16 months, four visits for those aged 13 to <16 months, five visits for ages 12 to <13 months, seven visits for ages 3 to <12 months, and six visits for ages <3 months.

Maternal recall was assessed across 20 ANC messages, 13 birth-related messages, and 18 postpartum care messages. We calculated Cronbach’s alpha to assess the internal consistency of responses and computed the number of correctly reported messages within each phase to generate a composite recall score.

We administered the Edinburgh Postnatal Depression Scale (EPDS), a self-reported questionnaire validated in the Philippines, to assess the presence and severity of postpartum depression [20,21]. It comprises 10 items that assess a mother’s emotional and psychological state during the previous week. Each item is scored on a four-point Likert scale ranging from 0 to 3, with higher scores indicating greater severity of depressive symptoms. The total score can range from 0 to 30, with EPDS scores ≥10 indicating possible depression and scores ≥13 suggesting a high likelihood of depression.

Dietary diversity score (DDS) for women represents a numerical indicator of 10 food groups consumed by participants (grains, pulses, nuts and seeds, milk and milk products, flesh, eggs, dark green leafy vegetables, vitamin A-rich fruits and vegetables, and other fruits and vegetables) [22]. Minimum dietary diversity (MDD) for women was defined as a percentage of women who consumed at least 5 out of 10 food groups in the past day (≥5 dietary diversity score for women).

Child dietary diversity was understood as an MDD defined as consumption of five out of eight food groups during the previous day [23]. The seven food groups were grains, pulses, vitamin A-rich fruits and vegetables, other fruits and vegetables, flesh foods (*i.e*. meat, organ meat, or fish/seafoods), dairy products, and eggs.

Child nutritional status was determined by calculating the length-for-age Z-score (LAZ), weight-for-age Z-score (WAZ), and weight-for-length Z-score (WLZ) according to the WHO child growth standards [24]. We defined stunting as a LAZ score below −2, wasting as a WLZ score below −2, and underweight as a weight-for-age (WAZ) score below −2 [24].

Covariates included women’s age, age of the first pregnancy, number of pregnancies, marital status, occupation, highest education level, asset index, mobile phone ownership, health insurance, household wealth quintile [25], and household food security [26].

### Data collection and measurement

We developed paper-based questionnaires and transferred them to the Kobo toolbox, an online survey tool [27], while a PhD Filipino researcher translated them from English to Tagalog. This standardised questionnaire included the following sections: demographic and socioeconomic status; reproductive health practices; antenatal, delivery, and postnatal care; infant and young child feeding; maternal mental health; child’s dietary diversity; BHW’s home visit counselling. Child weight was measured with HUS-316B digital weighing scales (HuBDIC, Korea), length with an infant measure mat, and mid-upper arm circumference (MUAC) with a non-stretchable tape measure placed around the midpoint of the upper arm. All measurements were recorded to the nearest 0.1 cm.

A Manila-based nonprofit research organisation recruited two supervisors and 24 local enumerators fluent in the local language and living in Eastern Visayas. Researchers provided enumerators with a three-day training session in Tacloban City and a day pilot test in the non-study Barangay.

We generated a household listing of eligible participants of pregnant women and mothers with children 0–11.9 months of age in both sites, after which we randomly selected the final sample and shared the list with enumerators prior to data collection. Enumerators then conducted household visits to assess eligibility and obtained informed consent. The first round of data collection lasted from 25 September to 12 October 2023, while the second round of visits was conducted from 16 September to 3 October 2024.

### Data analysis

We analysed data according to the intention-to-treat principle to ensure that all participants were included in the groups to which they were originally assigned. We compared the sociodemographic characteristics of the intervention and comparison groups, as well as BHW’s household visits at one-year follow-up, using χ^2^ tests for categorical variables and Student’s *t* tests for continuous variables. We used generalised linear regression models to estimate the programme’s impact on maternal message recalls related to pregnancy, birth, and postpartum care and children’s DDS and MDD (aged ≥6 months) at follow-up. We also used multi-level mixed-effects linear models to estimate its effect on women’s mental health and dietary diversity, and children’s nutritional status. We specified fixed effects as the programme exposure (*vs*. comparison), while random effects comprised the individual subject and Barangay locations. Interaction terms between time to assessment (enrolment *vs*. follow-up) and the programme exposure were included, indicating whether the effect of the intervention differs at different times. Enrolment differences in socioeconomic and demographic factors (*i.e.* religion, health insurance), maternal education, and household food security status were adjusted in the analysis. We assessed multicollinearity using the variance inflation factor, with all values ranging from 1 to 2.2, indicating no significant multicollinearity. We reviewed the normality of continuous variables using Q-Q plots (‘qnorm’ command). Model fit was examined using the Akaike information criterion and the Bayesian information criterion across different specifications. Mixed effect models including food security showed lower Akaike information criterion values compared to models that had excluded it.

We analysed all data Stata, version 17.0 (StataCorp, College Station, TX, USA).

## RESULTS

**Figure 2:**
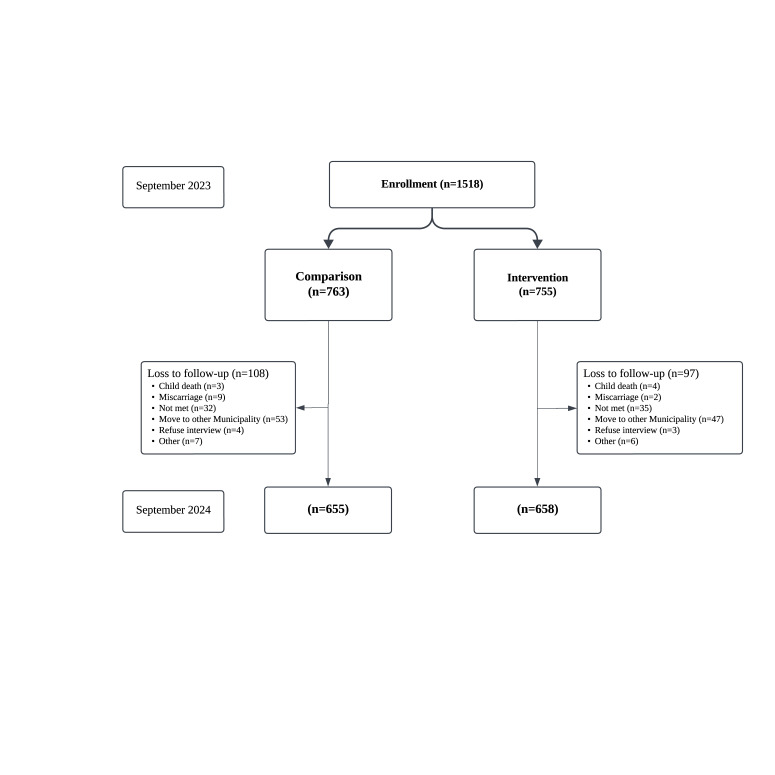
Study flow.

We enrolled 1518 women in the study in September 2023 – 763 in the comparison and 755 in the intervention group (**Figure 2**), with 202 and 192 being pregnant, respectively. After a year, 1313 (655 (85.8%) in the comparison group and 658 (87.2%) in the intervention group) were followed up in September 2024. The reasons for loss to follow-up included child death (n = 3 in comparison, n = 4 in intervention), miscarriage (n = 9 in comparison, n = 2 in intervention), not met (n = 32 in comparison, n = 35 in intervention), moved to other places (n = 2 in comparison, n = 2 in intervention), refused interview (n = 4 in comparison, n = 4 in intervention), and other reasons (n = 7 in comparison, n = 7 in intervention).

Most socioeconomic and demographic characteristics were comparable between intervention and comparison areas (**Table 1**). Almost all study participants were of the Waray tribe (97.2%) and were Roman Catholic (96.0%). The mean family size was 5.5 (standard deviation (SD) = 2.2), and 22.7% of mothers reported severe food insecurity. Educational attainment was relatively high, with 55.6% of women having completed secondary school and 28.5% exceeding the level. The average age of women at enrolment was 28 years (SD = 6.0). The proportion of women using PhilHealth insurance was higher in the comparison group than in the intervention group (51.4% *vs*. 40.0%; *P* < 0.001). The mean child age (n = 919) was 6.4 months (SD = 3.5). Enrolment characteristics did not differ significantly between women retained in the study and those lost to follow-up, although those lost to follow-up were more likely to be younger, from wealthier households, have higher internet use, and be at an earlier gestational age at enrolment (Table S2 in the **Online Supplementary Document**).

**Table 1:** Sociodemographic and economic characteristics at enrolment, September to October 2023 (n = 1518)*

	Comparison	Intervention	*P*-value†
**Household-level**	n = 763	n = 755	
Religion			0.02
*Roman Catholic*	741 (97.1)	716 (94.8)	
*Other*	22 (2.9)	39 (5.2)	
Ethnicity			0.42
*Waray*	744 (97.5)	733 (96.8)	
*Other*	19 (2.5)	24 (3.2)	
Family size, x̄ (SD)	5.5 (2.2)	5.5 (2.3)	0.86
Use of electricity	737 (96.6)	719 (95.2)	0.18
Wealth quintile‡			0.20
*Poorest*	148 (19.7)	152 (20.4)	
*Poor*	166 (22.1)	168 (22.6)	
*Middle*	145 (19.3)	125 (16.8)	
*Richer*	134 (17.8)	163 (21.9)	
*Richest*	159 (21.1)	137 (18.4)	
*Missing*	11	10	
Owned mobile phone	649 (85.1)	649 (86.0)	0.62
Household food security§			0.56
*Secure*	240 (31.5)	223 (29.5)	
*Mildly insecure*	194 (25.4)	179 (23.7)	
*Moderately insecure*	169 (22.1)	184 (24.4)	
*Severe*	160 (21.0)	169 (22.4)	
**Maternal level**	n = 763	n = 755	
Age in years, x̄ (SD)	28.1 (6.3)	27.8 (6.7)	0.32
Highest education attainment			0.09
*Primary*	112 (14.7)	123 (16.3)	
*Secondary*	414 (54.3)	436 (57.8)	
*More than secondary*	237 (31.1)	196 (26.0)	
Occupation			0.11
*Housewife*	645 (84.5)	637 (84.4)	
*Employee (government/private)*	45 (5.9)	61 (8.1)	
*Self-employed/entrepreneur/other*	73 (9.6)	57 (7.6)	
Health insurance			<0.001
*Any PhilHealth*	392 (51.4)	303 (40.0)	
*None*	364 (47.7)	440 (58.1)	
*Other*	7 (0.9)	14 (1.8)	
Marital status			0.86
*Cohabiting*	374 (49.0)	360 (47.7)	
*Married*	201 (26.3)	196 (26.0)	
*Single with partner*	174 (22.8)	182 (24.1)	
*Single without partner/widowed/divorced*	14 (1.8)	17 (2.2)	
Listen to the radio			0.34
*Not at all*	499 (65.4)	482 (63.8)	
*Less than once a week*	136 (17.8)	125 (16.6)	
*At least once a week*	128 (16.8)	148 (19.6)	
Watch television			0.91
*Not at all*	270 (35.4)	274 (36.3)	
*Less than once a week*	180 (23.6)	179 (23.7)	
*At least once a week*	313 (41.0)	302 (40.0)	
Use of internet			0.62
*Almost everyday*	402 (52.7)	379 (50.2)	
*At least once a week*	186 (24.4)	192 (25.4)	
*Less than once a week*	175 (22.9)	184 (24.4)	
**Child level¶**	n = 517	n = 532	
Sex			0.76
*Male*	272 (52.6)	285 (53.6)	
*Female*	245 (47.4)	247 (46.4)	
Age in months, x̄ (SD)	6.4 (3.5)	6.3 (3.4)	0.81

SD – standard deviation, x̄ – mean

^*^Presented as n (%) unless specified otherwise.

†*P*-values are based on χ^2^ test for categorical variables and Student’s *t* test for continuous variables.

‡Wealth quintiles were generated using principal component analysis, based on a list of assets that included electricity, radio, TV, landline, freezer, oven, stove, microwave, DVD player, karaoke machine, cable service, air conditioner, watch, mobile phone, computer, bicycle, tricycle, e-trike, animal-drawn cart, car, tractor, boat, improved water source, and improved toilet facility [25].

§Household food security was assessed by Household Food Insecurity Access Scale [26]

¶Child level information includes data on children aged 0 to less than 12 months at enrollment.

A total of 87.7% of women in the intervention group were ever visited by a BHW, while only 75.0% of women in the comparison group had BHW visits (*P* < 0.001). On average, women in the intervention group received 4.4 visits (SD = 3.6) over the year compared to 2.5 visits (SD = 2.8) in the comparison group (*P* < 0.001). Nearly half of households (46.7%) received the recommended number of or more BHW home visits, while only 28.7% of households in the comparison areas had the expected home visits (*P* < 0.001). At each time point, the mean number of home visits by BHWs was higher in the intervention areas than the comparison areas, both during pregnancy (mean (x̄) = 3.2, SD = 2.7 vs x̄ = 1.7, SD = 2.0; *P* < 0.001) and from birth to six months postpartum (x̄ = 2.6, SD = 2.0 vs x̄ = 1.3, SD = 1.4; *P* < 0.001) (**Table 2**).

**Table 2:** Frequency and coverage of home visits by Barangay health workers at follow-up (n = 1313)*

	Comparison (n = 655)	Intervention (n = 658)	*P*-value†
**Ever visited by BHW, n (%)**	491 (75.0)	577 (87.7)	<0.001
**Number of BHW visits over the year**	2.5 (2.8)	4.4 (3.6)	<0.001
**Adherence of BHW visits over the year, n (%)‡**	n = 645	n = 619	<0.001
0%	173 (26.8)	82 (13.3)	
0–50%	182 (28.2)	99 (16.0)	
50–75%	95 (14.7)	119 (19.2)	
75 to <100%	10 (1.6)	30 (4.8)	
≥100%	185 (28.7)	289 (46.7)	
BHW visits during pregnancy	1.7 (2.0)	3.2 (2.7)	<0.001
BHW visits from delivery to six months postpartum	1.3 (1.4)	2.6 (2.1)	<0.001
BHW visits after six months postpartum	1.3 (1.5)	2.4 (2.3)	<0.001
**BHW home visit information**	n = 491	n = 577	
Family member attendance during BHW's home visit, n (%)	188 (38.3)	280 (48.5)	0.001
Time spent by BHW during the most recent home visit, n (%)			<0.001
*<5 min*	124 (25.2)	74 (12.8)	
*5–10 min*	207 (42.2)	202 (35.0)	
*10–20 min*	95 (19.3)	161 (27.9)	
*≥20 min*	65 (13.3)	140 (24.2)	

BHW – Barangay health worker

*Presented as mean (standard deviation) unless specified otherwise.

†*P*-values are based on χ^2^ test for categorical variables and Student’s *t* test for continuous variables.

‡The expected number of visits in the past year was as follows: three visits for children aged ≥16 months, four visits for those aged 13 to <16 months, five visits for ages 12 to <13 months, seven visits for ages 3 to <12 months, and six visits for ages <3 months.

**Figure 3:**
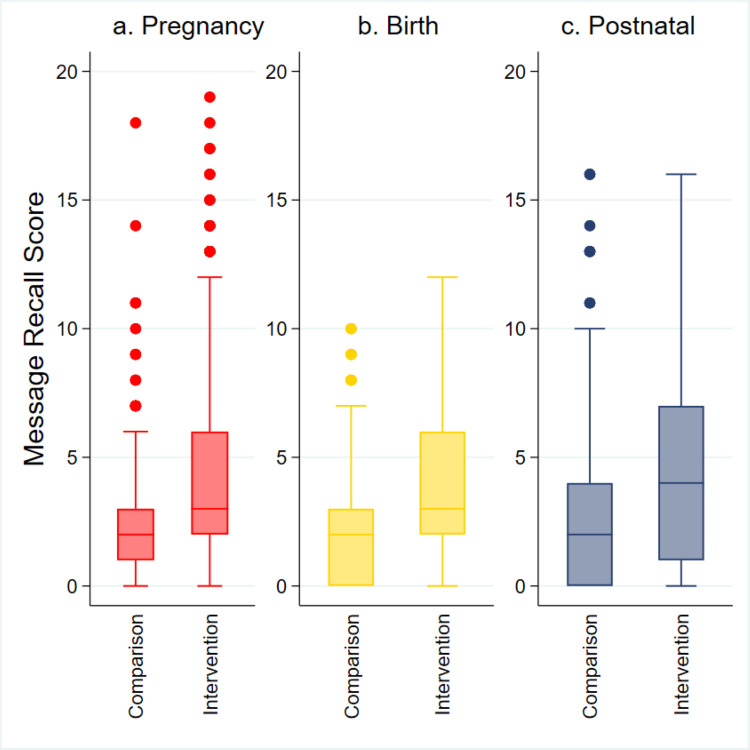
Boxplots of messages recall scores related to pregnancy, birth, and postnatal care among mothers visited by BHWs (n = 491 in comparison, n = 591 in intervention). **Panel A.** Pregnancy message recall. **Panel B.** Birth message recall. **Panel C.** Postnatal message recall.

The BHWs in the intervention areas spent more time in the study participants’ households: over half (51.1%) of their most recent visits lasted longer than 10 minutes, compared to only 32.6% in the comparison areas (*P* < 0.001). The presence of family members during BHW visits was more common in the intervention areas (48.5% *vs*. 38.3%; *P* < 0.001). Women in the intervention group had significantly higher recall scores for pregnancy-related messages compared to those in the comparison group (x̄ = 4.4, SD = 3.8 *vs*. x̄ = 2.2, SD = 2.2; *P* < 0.001). Similarly, recall scores for birth-related messages (x̄ = 3.9, SD = 3.1 *vs*. x̄ = 2.1, SD = 2.1; *P* < 0.001) and postnatal messages (x̄ = 4.7, SD = 3.9 *vs*. x̄ = 2.6, SD = 2.9; *P* < 0.001) were also significantly higher among women in the intervention group (**Figure 3****;** Tables S3, S4a, and S4b in the **Online Supplementary Document**).

The mean EPDS score (x̄ = 8.6, SD = 5.4 *vs*. 8.9, SD = 5.6) and the proportion of possible depressive symptoms (49.2% *vs*. 46.3%) were similar between the intervention and comparison groups at enrolment (**Table 3**). The proportion of maternal possible depressive symptoms in the intervention group showed no notable changes after a year of follow-up, while the proportion increased from 49.2% at enrolment to 60.0% in comparison areas a year later. After adjusting for enrolment differences in EPDS or the percentage of potential depressive symptoms, we found that the intervention reduced the proportion of potential depressive symptoms by 9.16 percentage points (pp) (95% confidence interval (CI) = −15.7, 2.62) and the EPDS score by −1.20 (95% CI = −1.88, −0.52). However, there was no programme impact on reducing severe depression. The changes in EPDS were associated with duration of household visits, with women who received visits lasting at least 20 minutes showing greater improvements with a change of −3.6 points in the EPDS score (95% CI = −6.1, −1.0), compared to those whose visits lasted less than five minutes. We found no such association in the comparison group (Table S5 in the **Online Supplementary Document**).

**Table 3:** Impact of ttCF programme on dietary diversity and depression among study mothers, Eastern Visayas, Philippines

	Comparison		Intervention			
**Indicators**	**Enrolment (n = 763)**	**Follow-up (n = 655)**	**Enrolment (n = 755)**	**Follow-up (n = 658)**	Unadjusted impact size (95% CI)*	**Adjusted impact size, pp (95% CI)**†
**Maternal level**						
Maternal mental health						
*EPDS, x̄ (SD)*	8.9 (5.6)	9.0 (5.8)	8.6 (5.4)	7.8 (6.1)	−1.29 (−1.87, −0.52)	−1.20 (−1.88, −0.52)
*Possible depression (EPDS≥10), n (%)*	375 (49.2)	458 (60.0)	458 (46.3)	363 (48.1)	−9.20 (−15.7, −2.6)	−9.16 (−15.7, −2.62)
*Moderate/severe depression (EPDS≥13), n (%)*	164 (21.5)	146 (19.1)	153 (20.3)	138 (18.3)	0.17 (−5.7, 5.4)	−0.23 (−5.77, 5.30)
Maternal diet						
*Dietary diversity Score, x̄ (SD)‡*	4.9 (2.1)	3.9 (2.2)	5.4 (2.3)	4.0 (2.0)	−0.48 (−0.73, −0.24)	−0.48 (−0.73, −0.23)
*Minimum dietary diversity, n (%)§*	400 (52.4)	290 (38.0)	464 (61.4)	309 (40.9)	−6.55 (−12.50, −0.13)	−6.49 (−12.91, 0.00)
						
**Child level**						
LAZ, x̄ (SD)¶	−0.25 (1.42)	−1.17 (1.25)	−0.28 (1.40)	−1.15 (1.10)	0.06 (−0.11, 0.23)	0.08 (−0.09, 0.25)
WLZ, x̄ (SD)║	−0.86 (1.45)	−0.91 (1.28)	−0.71 (1.43)	−0.65 (1.32)	0.08 (−0.13, 0.30)	0.09 (−0.13, 0.30)
WAZ, x̄ (SD)**	−0.84 (1.33)	−1.20 (1.17)	−0.76 (1.40)	−1.07 (1.22)	0.06 (−0.11, 0.23)	0.07 (−0.10, 0.24)
MUAC in cm, x̄ (SD)†	13.79 (1.61)	14.80 (1.16)	14.00 (1.71)	14.95 (1.15)	−0.04 (−0.25, 0.16)	−0.10 (−0.30, 0.11)
Stunting (LAZ<−2), n (%)¶	48 (9.4)	114 (25.7)	49 (9.3)	99 (21.5)	−4.00 (−9.56, 1.54)	−4.46 (−10.00, 1.09)
Wasted (WLZ<−2), n (%)║	99 (19.6)	87 (19.8)	79 (15.4)	61 (13.7)	−0.84 (−6.85, 5.18)	−0.95 (−6.97, 5.07)
Underweight (WAZ<−2), n (%)**	85 (16.5)	107 (23.7)	86 (16.4)	96 (20.9)	−2.37 (−8.13, 3.39)	−2.84 (−8.60, 2.91)

CI – confidence interval, EPDS – Edinburgh Postnatal Depression Scale, LAZ – length-for-age Z-score, MUAC – mid-upper arm circumference, pp – percentage point, SD – standard deviation, WAZ – weight-for-age Z-score, WLZ – weight-for-length Z-score, x̄ – mean

*For maternal level analysis, multi-level mixed-effects linear models were employed to estimate the programme’s impact (β, 95% CI). Programme exposure was included as a fixed effect, while individual participants and Barangay locations were treated as random effects. Interaction terms between time to assessment and program exposure were included to present time-dependent effects. For child-level analysis, all models were adjusted for child age, child sex, health insurance, religion, maternal education, and household food security.

†Adjusted for health insurance, religion, maternal age, maternal education, and household food security

‡Dietary diversity score represents the number of food groups consumed by participants

§Minimum dietary diversity was defined as consumption of at least 5 out of 10 food groups (≥5 DDS).

¶LAZ and stunting: n = 511 for comparison and n = 526 for intervention at enrolment; n = 448; n = 448 for comparison and n = 462 for intervention at follow-up.

║WLZ and wasting: n = 505 for comparison and n = 511 for intervention at enrolment; n = 448 for comparison and n = 462 for intervention at follow-up.

**WAZ and underweight: n = 514 for comparison and n = 523 for intervention at enrolment; n = 452 for comparison and n = 464 for intervention at follow-up.

†MUAC: n  = 516 for comparison and n = 527 for intervention at enrolment; n = 429 for comparison and n = 445 for intervention at follow-up.

At enrolment, a higher proportion of women in the intervention group met the MDD criteria compared to those in the comparison group (61.4% *vs*. 52.4%; *P* < 0.001) (**Table 3**). One year later, the proportion of women meeting MDD decreased in both groups (40.9% vs 38.9%). After a year of follow-up, there was a significant reduction in the DDS score (−0.48; 95% CI = −0.73, −0.23) or in the proportion of MDD (–6.49 pp; 95% CI = −12.9, 0.00) in the intervention areas.

There were no significant differences between the intervention and comparison areas on DDS (3.7 *vs*. 3.6; adjusted impact size = −0.12; 95% CI = −0.40, 0.16) or MDD (28.0% *vs*. 30.7%; adjusted risk ratio = 0.88; 95% CI = 0.69, 1.13) among children aged ≥6 months (Table S6 in the **Online Supplementary Document**). At enrolment, the prevalence of stunting (9.3% *vs*. 9.4%), wasting (15.4% *vs*. 19.6%), and underweight (16.4% *vs*. 16.4%) among children was similar across groups. After one year of intervention, there was no significant impact on the reduction of stunting (adjusted impact size = −4.46 pp; 95% CI = −10.0, 1.09), wasting (−0.95 pp; 95% CI = −6.97, 5.07), and underweight (−2.84 pp; 95% CI = −8.60, 2.91) after accounting for enrolment differences (**Table 3****)**.

## DISCUSSION

We evaluated the impact of timed and targeted home visit counselling by BHWs on the frequency and duration of visits, message recall across the prenatal to postpartum periods, maternal mental health, and maternal and child diet and nutrition outcomes in Eastern Visayas, Philippines. We found that BHWs in the intervention areas made significantly more frequent home visits during the first 1000 days, had a longer duration of visit, and involved more family members during visits. Women in the intervention group were able to recall a higher proportion of messages related to the care for the first 1000 days of life. Notably, timed and targeted home visits improved maternal mental health; however, there was no impact on diet and nutritional status among women and children.

Although the role of BHWs is essential in promoting healthy activities in the Philippines communities, many BHWs lack opportunities to get professional training and have received inconsistent and insufficient support and resources. The BHWs in rural areas often face poorer treatment compared to their urban counterparts [11,28]. To date, no studies have intensively evaluated the frequency, adherence to, and duration of home visits by CHWs, with most evaluations reporting on directly measured intended outcomes, without providing insights into the pathways through which home visits lead to the programme impact. Our study filled such gaps by examining household visits, message recall, and expected outcomes.

Compared to the comparison areas, households in the intervention areas in our study received a greater number of visits (2.7 *vs*. 4.5), were more likely to have a timely and recommended number of visits (28.7% *vs*. 46.7%) and experienced longer visit durations of 20 minutes or longer during the one-year follow-up period (13.3% *vs*. 24.2%). Notably, mothers in the intervention areas demonstrated significantly better recall of key messages.

A mental health study in the Philippines reported 16.4% of postnatal depression, indicating a significant mental health burden in the country [20]. To the best of our knowledge, this is the first study in the Philippines to demonstrate the potential of improving postpartum mental health through community-based home visits. The timed and targeted visits by BHWs reduced possible depressive symptoms by 9%, though not in severe depressive conditions. The improvement in EPDS during follow-up was associated with time spent during household visits, only among women who reported at least 20 minutes. This suggests that BHWs trained by ttCF may have offered greater psychological support to mothers spending enough interactive time. In practice, the ttCF programme provided training to BHWs for identifying at least three signs of maternal mental or psychosocial issues, using psychological first aid to support distressed mothers, and positive and negative coping strategies for mental well-being (Figure S1 in the **Online Supplementary Document**). Home visits can provide mothers with a comfortable and private space for emotional support and discussions of concerns [29]. Until now, however, the detection, screening, and treatment services for postnatal depression remain underdeveloped within the primary health care system in the Philippines.

Our study adds evidence to existing similar studies. A meta-analysis suggested that streamlined psychological interventions, when delivered by affordable and widely available CHWs, can reduce common mental disorders in adult populations [30]. Another recent systematic review indicated that interpersonal psychotherapy is an effective treatment to reduce postpartum depressive symptoms in low- and middle-income countries [31]. One randomised controlled trial in rural Tanzania reported that a CHW-delivered integrated intervention significantly reduced maternal depressive symptoms [32], while another conducted in Pakistan showed that a psychological intervention was effective in reducing depression among prenatally depressed women [33]. Findings from the WHO-endorsed Thinking Healthy Programme reported improved maternal functioning but did not significantly reduce severe perinatal depression symptoms [34].

In the future, refresher training and counselling skill-building are essential to amplify the programme’s impact, especially on mental health in Eastern Visayas. Our findings support the idea that BHWs trained with communication skills and psychological first aid may respond to mothers in distress appropriately, providing emotional support and knowledge acquisition.

Our ttCF programme showed no improvements in diet or nutrition outcomes among women and children. First, recall of the 20 counselling messages was poor, with only a few participants scoring above 30%. The recall for child feeding messages, such as adequate meal frequency for age (4.1%) and optimal complementary feeding (16.0%) was poor (Table S4b in the **Online Supplementary Document**). These findings suggest the need for prioritising key messages such as breastfeeding and complementary feeding for better retention, improvement in counselling techniques to ensure more effective communication, regular refresher trainings, and spending at least 20 minutes on home visit counselling.

Second, poor households might have difficulties in affording a variety of healthy food ingredients, as about 20% of study participants reported severe food insecurity (**Table 1**). Women’s mental health outcomes may be more responsive to the frequency of psychosocial support and interpersonal interaction between BHWs and women, while changes in diet and nutritional status are more structurally constrained by food access. It is recommended that the ttCF programme is coordinated with the agriculture and social protection sectors to improve food access for vulnerable communities.

Third, comparison areas received food supplementation such as Vita Meena, as reported by 42.3% of mothers with children. Compared to children without nutrient supplements in the comparison areas, those who received nutrient supplements in the comparison areas showed a higher increase in LAZ, WAZ, or WLZ, although this difference was not significant (Table S7 in the **Online Supplementary Document**). In some comparison areas also benefited from the Philippine Multisectoral Nutrition Project.

### Strengths and limitations

We used a quasi-experimental design with a large sample size, followed participants for a year, and assessed the changes in multiple health outcomes related to the first 1000 days of life. We also tracked the frequency, duration, and content of BHW home visits, providing in-depth insight into the programme pathways. However, several limitations need to be kept in mind. First, the ttCF intervention allocation was not randomised, which limited our ability to infer causality and introduced potential selection bias. Although comparability was achieved at the demographic and socioeconomic level, there may be unmeasured confounders such as capacity and service quality of local health facilities and geographically isolated and disadvantaged areas. In future scale-ups, a cluster- randomised trial or stepped wedge design may provide better rigour and strength of evidence. Second, there might be possible recall and reporting bias regarding dietary diversity, BHW home visits, and ttCF message recall. Women in the intervention areas might be subject to social desirability bias and interviewer effects, as the MNCH project is ongoing. The maternal and child diet was based on maternal recall rather than direct observation. Third, external nutrition interventions in the comparison, particularly food supplementation for children, might dilute the programme’s impact on diet and nutritional status. Fourth, a one-year follow-up may not be sufficient time for ttCF programme to detect meaningful changes in child nutritional status (*e.g.* LAZ/WAZ), given that the full ttCF visit schedule spans 1000 days from early pregnancy to 24 months of age. Lastly, the generalisability of these findings may be limited to rural Eastern Visayas or similar contexts, predominantly inhabited by households of the Waray ethnic group and the Roman Catholic faith.

## CONCLUSIONS

Using a quasi-experimental design, we evaluated the ttCF programme’s impact on maternal and child health and nutrition during the first 1000 days of life through home visits by trained BHWs. While ttCF significantly improved BHW home visit frequency and reduced maternal depressive symptoms, it had no effect on maternal diet or child nutrition outcomes. Aligned with the Magna Carta of Barangay Health Workers [28], the ttCF programme is promising to professionalise BHWs and strengthen the community health system. To ensure sustainability, our study findings underscore the need to institutionalise and reinforce the role of BHWs in providing mental health support to pregnant and postpartum women. Given the harsh weather conditions and limited access to geographically isolated areas, we recommend that the local government at the RHU and regional levels develop a comprehensive exit plan following the MNCH project. This may include monetary or non-monetary incentives, formal certifications and a strengthened supervision mechanism. Further study is needed to assess the extent of family members and community engagement, and local government efforts in sustaining the intervention.

## Additional material


Online Supplementary Document
