# Supplementary material for: Impact of home-visit counselling on maternal and child health and nutrition by Barangay health workers: a quasi-experimental study from Eastern Visayas, Philippines
Source: J Glob Health. 2025 Sep 26;15:04276. doi: 10.7189/jogh.15.04276 (PMC12475952; doi:10.7189/jogh.15.04276)
Supplement: Online Supplementary Document [file jogh-15-04276-s001.pdf]

Supplement to: Kang Y, Choi A, Kim H, Edward A, Ji H, Jeon J. Impact of home-visit counselling on maternal and child health and nutrition by Barangay health workers: a quasi-experimental study from Eastern Visayas, Philippines. J Glob Health. 2025;15:04276.

Supplemental Table 1. Key messages of a timed and targeted care for families (ttCF) program by time of visit

Checklist	Pregnancy				Essential newborn and postnatal care				Children 0-24 months			
	V1	V2	V3	V4	V5	V6	V7	V8	V9	V10	V11	V12
	2-4 m	4-6 m	7-8 m	8-9 m	0-7d	1m	3m	5m	8m	12 m	18 m	24 m
Additional serving	v	v	v	v								
Balanced and diverse meal	v	v	v	v								
Iodized salt	v	v	v	v								
Iodine capsule	v	v	v	v								
Iron rich diet	v	v	v	v								
Iron and folic acid	v	v	v	v								
Calcium Carbonate		v	v	v								
ANC visits	v	v	v	v								
Blood Typing	v											
Birth plan	v	v	v	v								
Mosquito net used regularly	v	v	v	v								
Handwashing with soap	v	v	v	v								
Access to improved latrine	v	v	v	v								
Deworming (once only)			v	v								
Hemoglobin Count	v											
Hematocrit Count	v											
Gestational Diabetes Screening	v											
Hepatitis B Screening	v											
HIV testing	v											
Syphilis Screening	v											
Danger Signs	v	v	v	v								
Facility birth					v							
Skilled attendant					v							
Family planning				v	v	v						
Iron and folic acid				v	v	v						
Balanced & diverse meal				v	v	v						
Postnatal consultation				v	v	v						
Maternal hygiene				v	v	v						
Water stored safe & ready				v	v	v						

Access to improved latrine	v	v	v						
Rest & family support	v	v	v						
Partner/family participation	v	v	v						
Danger signs	v	v	v						
Calcium Carbonate	v	v	v						
Essential newborn care		v	v						
Care of the cord		v							
Vaccination (Birth dose of BCG)		v	v						
Vaccination (Birth dose of Hepatitis B)		v	v						
Mosquito net used regularly		v	v						
Exclusive breastfeeding		v	v						
Routine check-up		v	v						
Routine check-up		v	v						
Danger signs surveillance		v	v						
Keeping the baby warm		v	v						
Handwashing before touching the baby		v	v						
Clean safe home & play things		v	v						
Partner/Family participation		v	v						
OPV Vaccination completed			v	v	v	v	v	v	v
Pentavalent Vaccination completed			v	v	v	v	v	v	v
PCV Vaccination completed			v	v	v	v	v	v	v
IPV Vaccination completed			v	v	v	v	v	v	v
MMR Vaccination completed			v	v	v	v	v	v	v
Growth monitoring			v	v	v	v	v	v	v
Continued breastfeeding			v	v	v	v	v	v	v
Mosquito net used regularly			v	v	v	v	v	v	v
Play and communication			v	v	v	v	v	v	v
Birth Registration			v	v	v	v	v	v	v
Diet diversity			v	v	v	v	v	v	v
Adequate meal frequency for age			v	v	v	v	v	v	v
Complementary feeding			v	v	v	v	v	v	v
Vitamin A			v	v	v	v	v	v	v
MUAC			v	v	v	v	v	v	v
Safe drinking water			v	v	v	v	v	v	v
Deworming			v	v	v	v	v	v	v
Partner/family participation			v	v	v	v	v	v	v

Supplemental Table 2. Comparison of socio-economic and demographic characteristics between analytic samples (n=1,313) and loss to follow-up (n=205)

Characteristics	Loss to follow-up (n=205)	Study included (n=1,313)	*P*-value*
	n (%)	n (%)	
Household level	n=763	n=755	
Religion			0.10
Roman Catholic	201 (98.0)	1,256 (95.7)	
Other	4 (2.0)	57 (4.3)	
Ethnicity			0.15
Waray	196 (95.6)	1,279 (97.4)	
Other	9 (4.4)	34 (2.6)	
Family size, Mean (SD)	5.5 (2.3)	5.5 (2.2)	0.90
Use of Electricity	194 (94.6)	1,262 (96.1)	0.32
Wealth quintile†			0.26
Poorest	43 (21.1)	257 (19.9)	
Poor	37 (18.1)	297 (23.0)	
Middle	40 (19.6)	230 (17.8)	
Richer	35 (17.2)	262 (20.3)	
Richest	49 (24.0)	247 (19.1)	
Owned mobile phone	173 (84.4)	1,125 (85.7)	0.63
Household Food Security‡			<0.001
Secure	91 (44.4)	372 (28.3)	
Mildly insecure	45 (22.0)	328 (25.0)	
Moderately insecure	40 (19.5)	313 (23.8)	
Severe	29 (14.1)	300 (22.8)	
Maternal level			
Age (years), Mean (SD)	26.8 (6.3)	28.2 (6.5)	0.004
Highest level of school			0.08
Primary	21 (10.2)	214 (16.3)	
Secondary	124 (60.5)	726 (55.3)	
More than secondary	60 (29.3)	373 (28.4)	
Occupation			0.43
Housewife	11 (5.4)	95 (7.2)	
Employee (government/private)	173 (84.4)	1,109 (84.5)	
Self-employed/Entrepreneur/other	21 (10.2)	109 (8.3)	
Health insurance			<0.67
Any PhilHealth	90 (43.9)	604 (46.0)	
None	111 (54.1)	692 (52.7)	

Other	4 (1.9)	17 (1.3)	
Marital status			0.26
Cohabiting	111 (54.1)	623 (47.5	
Married	49 (239)	348 (26.5)	
Single, with partner	43 (21.0)	313 (23.8)	
Single without partner/widowed/divorced	2 (1.0)	29 (2.2)	
Listen to the radio			0.11
Not at all	48 (23.4)	228 (17.4)	
Less than once a week	34 (16.6)	227 (17.3)	
At least once a week	123 (60.0)	858 (65.3)	
Watch television			0.29
Not at all	73 (35.6)	542 (41.3)	
Less than once a week	54 (26.3)	305 (23.2)	
At least once a week	78 (38.1)	466 (35.5)	
Use of internet			0.01
Almost everyday	117 (57.1)	664 (50.6)	
At least once a week	34 (16.6)	344 (26.2)	
Less than once a week	54 (26.3)	305 (23.2)	

*P-values are based on chi-square test for categorical variables and student t-test for continuous variables.

†Wealth quintiles were categorized as follows: poorest (first quintile), poor (second quintile), middle (third quintile), richer (fourth quintile), and richest (fifth quintile), excluding 22 participants with missing data.

‡Household food security was assessed by Household Food Insecurity Access Scale [31]

Supplemental Table 3. Messages recall scores among mothers ever visited by BHWs

Program Impact	Comparison (n=610) Mean (SD)	Intervention (n=620) Mean (SD)	β-coefficient (95% CI)*	β-coefficient (95% CI) †
Pregnancy recall score	2.2 (2.2)	4.4 (3.8)	2.21 (1.51, 2.92)	2.25 (1.57, 2.94)
Birth recall score	2.1 (2.1)	3.9 (3.1)	1.79 (1.19, 2.40)	1.83 (1.24, 2.41)
Postnatal recall score	2.6 (2.9)	4.7 (3.9)	2.17 (1.39, 2.95)	2.20 (1.43, 2.96)

* Generalized linear regression models were used to derive the difference in the indicators as b-coefficient (95%CI)

†Adjusted for health insurance, religion, maternal age, and maternal education.

Supplemental Table 4a. Messages mentioned by study participants who were ever visited by BHW during pregnancy

Characteristics	Comparison (n=655)	Intervention (n=658)	*P*-value*
	n (%)	n (%)	
Add serving	115 (17.6)	221 (33.6)	0.01
Nutritious diet	129 (19.7)	478 (42.3)	<0.001
Iron-rich diet	107 (16.3)	227 (34.5)	<0.001
Iodized salt	18 (2.8)	68 (10.3)	<0.001
Iron and Folic Acid	158 (24.1)	252 (38.3)	<0.001
Calcium supplement	51 (7.8)	135 (20.5)	<0.001
ANC visits	181 (27.6)	292 (44.4)	<0.001
Deworming	8 (1.2)	33 (5.0)	<0.001
HIV test	15 (2.3)	37 (5.6)	0.002
Syphilis test	2 (0.3)	14 (2.1)	0.003
Tetanus vaccination	53 (8.1)	184 (28.0)	<0.001
Birth planning	70 (10.7)	145 (22.0)	<0.001
Use of mosquito next	26 (4.0)	119 (18.1)	<0.001
Hand washing	64 (9.8)	196 (29.8)	<0.001
Use of hygienic latrine	18 (2.8)	75 (11.4)	<0.001
Danger signs	74 (11.3)	135 (20.5)	<0.001

**P*-values are based on chi-square test for categorical variables.

Supplemental Table 4b. Birth to Postnatal messages remembering ttCF messages from BHW

Characteristics	Comparison (n=655)	Intervention (n=658)	*P*-value*
	n (%)	n (%)	
Essential newborn care	252 (38.5)	345 (52.4)	<0.001
Care of the cord	80 (12.2)	189 (28.7)	<0.001
Vaccination (Birth dose of BCG)	101 (15.4)	236 (35.9)	<0.001
Vaccination (Birth dose of Hepatitis B)	79 (12.1)	194 (29.5)	<0.001
Mosquito net used regularly	28 (4.3)	143 (21.7)	<0.001
Exclusive breastfeeding	151 (23.0)	306 (46.5)	<0.001
Routine check-up	169 (25.8)	245 (37.2)	<0.001
Play and communication	19 (2.9)	81 (12.3)	<0.001
Danger signs surveillance	59 (9.0)	109 (16.6)	<0.001
Keeping the baby warm	38 (5.8)	80 (12.2)	<0.001
Handwashing before touching the baby	73 (11.1)	211 (32.1)	<0.001
Clean, safe home & playthings	53 (8.1)	127 (19.3)	<0.001
Partner/Family participation	20 (3.1)	67 (10.2)	<0.001
OPV Vaccination completed	132 (20.1)	308 (46.8)	<0.001
Pentavalent Vaccination completed	95 (14.5)	223 (33.9)	<0.001
PCV Vaccination completed	96 (14.7)	214 (32.5)	<0.001
IPV Vaccination completed	82 (12.5)	193 (29.3)	<0.001
MMR Vaccination completed	101 (15.4)	221 (33.6)	<0.001
Growth monitoring	163 (24.9)	343 (52.1)	<0.001
Continued breastfeeding	143 (21.8)	242 (36.8)	<0.001
Mosquito net used regularly	32 (4.9)	122 (18.5)	<0.001
Play and communication	23 (3.5)	88 (13.4)	<0.001
Birth Registration	55 (6.4)	71 (10.8)	0.14
Diet diversity	8 (1.2)	45 (6.8)	<0.001
Adequate meal frequency for age	10 (1.5)	27 (4.1)	0.01
Complementary feeding	36 (5.5)	105 (16.0)	<0.001
Vitamin A supplementation	141 (21.5)	244 (37.1)	<0.001
MUAC measurement	111 (17.0)	135 (20.5)	0.100
Safe drinking water	73 (11.2)	150 (22.8)	<0.001
Deworming	30 (4.6)	49 (7.4)	0.03
Partner/family participation	14 (2.1)	39 (5.9)	<0.001

**P*-values are based on chi-square test for categorical variables.

Supplemental Table 5. Association between change in EPDS from enrollment and one year follow-up and time spent by BHWs during household visits among women who were ever visited by Barangay Health Workers in the past year

	Comparison (n=491)		Intervention (n=577)	
Time spent by BHWs during household visits	Change in EPDS score from enrollment to one year follow-up, Points (95%CI)	p-val	Change in EPDS score from enrollment to one year follow-up, Points (95%CI)	p-val
<5 m	(REF)		(REF)	
5-<10 m	0.0 (-1.5, 1.6)	0.97	-1.3 (-3.7, 0.98)	0.25
10-<20 m	0.1 (-1.5, 1.8)	0.89	-1.4 (-4.0, 1.2)	0.30
≥20 m	-0.8 (-3.2, 1.5)	0.47	-3.6 (-6.1, -1.0)	0.01

Supplemental Table 6. Impact of timed and targeted care for family (ttCF) program on child feeding practices (aged ≥ 6 months) at follow-up

**Program Impact**	**Comparison (n=610)**	**Intervention (n=620)**	**Unadjusted b-coefficient (95% CI)** §	**Adjusted b-coefficient (95% CI)**†
Dietary diversity score‡, Mean (SD)	3.7 (1.7)	3.6 (1.5)	-0.07 (-0.37, 0.23)	-0.12 (-0.40, 0.16)
			**Unadjusted Relative Risk (RR) (95% CI)***	**Adjusted RR (95% CI)**†
Minimum dietary diversity¶, n (%)	190 (30.7)	175 (28.0)	0.91 (0.70, 1.18)	0.88 (0.69, 1.13)

* Generalized linear regression models were used to derive b-coefficient (95%CI)
† Adjusting health insurance, religion, maternal age, maternal education, household food security, and child age
‡ Dietary diversity score represents the number of food groups consumed by participants
§ Generalized linear regression models were used to derive relative risk (95%CI)
¶ Minimum dietary diversity defined as consumption of ≥ 4 out of 7 food groups including breastfeeding

Supplemental Table 7. Impact of timed and targeted care on nutrition outcomes: (1) Children in comparison group without nutrient supplementation; (2) Children in comparison group with nutrient Supplementation; and (3) intervention group

	Comparison group		
**Characteristics**	Children without nutrient supplementation	Children with nutrient supplementation	Intervention group
	**REF**	**β (95%CI)**	**β (95%CI)**
Length-for-age Z-score (LAZ)[‡]	-	0.47 (-0.06, 0.99)	-0.01(-0.18, 0.16)
Weight-for-length Z-score (WLZ) [§]	-	0.40 (-0.42, 1.23)	0.10 (-0.12, 0.32)
Weight-for-age Z-score (WAZ)[¶]	-	0.14 (-0.52, 0.80)	0.08 (-0.09, 0.25)
Mid-upper arm circumference[‖], cm	-	-0.19 (-0.98, 0.61)	-0.10 (-0.31, 0.11)
Stunting (LAZ<-2)[‡]	-	2.72 pp (-18.6, 24.1)	-4.25 pp (-9.85, 1.35)
Wasted (WLZ<-2)[§]	-	-1.97 pp (-25.0, 21.0)	-0.97 pp (-7.05, 5.10)
Underweight (WAZ<-2)[¶]	-	-1.59 pp (-38.2, 6.45)	-3.30 pp (-9.10, 2.51)

*Multi-level mixed-effects linear models were employed to estimate the program's impact (β, 95% CI). Program exposure was included as a fixed effect, while individual participants and Barangay locations were treated as random effects. Interaction terms between time to assessment and program exposure were included to present time-dependent effects

[†]Adjusted for child age, child sex, health insurance, religion, maternal education, and household food security

Supplemental Figure 1 Psychological First Aid outline in training manual of timed and targeted care for family (ttCF) program

PSYCHOLOGICAL FIRST AID: ACTION PRINCIPLES*

For responding to people in distress

LOOK

- Enquire / observe for safety*
- Enquire / observes for urgent basic needs (e.g. shelter/food/ health)*
- Enquire / observe for signs of distress

LISTEN

- Approach the person in a sensitive way to determine if they want support
- Listen without pressuring the person to talk
- If they wish to share, use good / sensitive listening skills
- Validate the person's concerns and assure confidentiality
- Explore the person's current coping strategies (both positive and negative)

LINK

- Encourage links with existing community and family support
- Link person to appropriate service provider if needed / accepted
- Explore ways the person can improve positive coping strategies, and give affirmation
- Help the person to feel calm

** If immediate danger or urgent needs identified, take action to protect the person from further harm. Assess if this is the appropriate time / place to proceed.*

PRACTICES TO BE AVOIDED

- ✗ Interrupting, poor listening
- ✗ Judgement, judgmental language
- ✗ Talking about own or someone else's problems
- ✗ Making false promises, false information or exaggerating skills
- ✗ Pressuring to take an action rather than respecting their wishes
